# Repeated genetic adaptation under demographic limits in kiwifruit

**DOI:** 10.1111/tpj.71163

**Published:** 2026-10-12

**Authors:** Mengyun Qin, Jingwen Huang, Yufang Shen, Quan Jiang, Na Wei, Xiaohong Yao

**Affiliations:** ^1^ State Key Laboratory of Plant Diversity and Specialty Crops, Wuhan Botanical Garden Chinese Academy of Sciences Wuhan Hubei 430074 China; ^2^ University of Chinese Academy of Sciences Beijing 100049 China

**Keywords:** inbreeding, mutation load, repeated adaptation, population genomics, *Actinidia hemsleyana*, *Actinidia eriantha*

## Abstract

Population genetic theory predicts that species with narrow geographic distributions experience stronger genetic drift, higher inbreeding, and greater mutational burden than their widespread relatives, yet how demographic history shapes the genomic basis of adaptation remains poorly understood. We hypothesized that (i) restricted‐range species exhibit reduced genetic diversity, elevated inbreeding, and greater mutation load owing to smaller long‐term effective population sizes; (ii) despite these demographic constraints, shared environmental pressures can still drive repeated adaptation; (iii) repeated adaptation may arise through ancestral standing genetic variation, adaptive introgression, or independent *de novo* mutations. We tested these hypotheses using two kiwifruit species, *Actinidia hemsleyana* (narrow‐ranged) and *Actinidia eriantha* (widespread), which differ markedly in demographic history and range size but occupy overlapping environments in subtropical China. We show that demographic contraction and inbreeding have eroded genome‐wide diversity in the range‐restricted species, accompanied by a substantially higher load of deleterious mutations. Despite these contrasting demographic legacies, both species exhibit repeated genomic responses to shared climatic gradients, with adaptation targeting similar genes. Such repeated adaptation was driven primarily by ancestral standing variation, with smaller contributions from adaptive introgression and only limited evidence for independent *de novo* mutations. These results reveal that demographic constraints shape genome‐wide diversity and mutation load but do not preclude repeated adaptation when shared genetic variation remains available.

## Significance Statement

Our study reveals that demographic constraints reshape the sources of adaptive variation but do not preclude repeated adaptation through standing genetic variation.

## INTRODUCTION

Genetic variation underpins evolutionary change, enabling populations to adapt and persist in changing environments. Population genetic theory predicts that demographic processes, such as population size, isolation, and historical bottlenecks, govern the balance between mutation, drift, and selection, thereby shaping the evolutionary potential of species (De Kort et al., 2021; Ellegren & Galtier, 2016; Hamrick et al., 1992; Salgotra & Chauhan, 2023). Species with small geographic ranges or small effective population sizes are therefore expected to harbor lower levels of genetic variation and higher inbreeding, exposing them to stronger genetic drift and an elevated burden of deleterious mutations (e.g., Feng et al., 2024; Liao et al., 2025; Yi et al., 2024). Yet, how these demographic limits translate into evolutionary outcomes remains incompletely understood. In particular, whether small, isolated populations can maintain or regain adaptive potential through purging of deleterious alleles or whether accumulated mutational load imposes lasting constraints on evolution remains a central unresolved question in evolutionary and conservation biology (Dussex et al., 2023; Grossen et al., 2020; Ma et al., 2021; Mathur & DeWoody, 2021; Peng et al., 2025; Poon & Otto, 2000; Robinson et al., 2018; Tao et al., 2024; Yang et al., 2024). Despite theoretical and empirical advances, we still lack a comparative understanding of how contrasting demographic histories translate into convergent or divergent evolutionary outcomes.

While demographic limits can erode genetic diversity and elevate mutation load, adaptation may nevertheless proceed along repeatable genomic paths when species experience similar selective pressures (Cerca, 2023; Moreira & Smith, 2023; Rellstab et al., 2020; van Boheemen & Hodgins, 2020). Such repeated adaptation provides a powerful framework for evaluating the predictability of evolution across lineages. At the genomic level, repeated adaptation is often reflected by the recurrent involvement of the same genes, loci, or pathways, suggesting that natural selection repeatedly targets similar genomic solutions to comparable environmental challenges (Chaturvedi et al., 2025). However, this pattern alone does not reveal the evolutionary origin of the adaptive variants. Similar genomic signatures of repeated adaptation can arise through several alternative evolutionary mechanisms, including (i) independent mutations, whereby beneficial mutations arise separately in each lineage after divergence; (ii) selection on ancestral standing variation, whereby the same pre‐existing alleles inherited from a common ancestor are repeatedly favored in different lineages; and (iii) adaptive introgression, whereby beneficial alleles spread among lineages through gene flow (Barrett & Schluter, 2008; Chaturvedi et al., 2025; Pease et al., 2016). Because these mechanisms can produce similar genomic signatures, distinguishing among them remains one of the central challenges in evolutionary genomics, particularly for closely related species with overlapping distributions and asymmetric demographic histories. Despite theoretical expectations that demographic constraints reduce the efficacy of selection and restrict adaptive potential (Chen et al., 2025), empirical tests of how demographic history modulates these alternative adaptive routes remain scarce. Understanding how demographic history, mutation load, and shared evolutionary ancestry jointly shape the genomic architecture of repeated adaptation is therefore essential for evaluating the repeatability and limits of evolution under demographic constraint.

Comparative population genomics provides a powerful framework for testing these theoretical predictions. When closely related species experience similar environments but differ markedly in demographic history, their genomes retain signatures of how drift, mutation load, and selection interact to shape adaptive evolution (Charlesworth, 2009; Peart et al., 2020; Xue et al., 2015). We hypothesized that species with smaller effective population sizes and narrower geographic ranges would exhibit lower genetic diversity, higher inbreeding, and a heavier mutational burden, consistent with stronger genetic drift and demographic contraction. Although these demographic constraints are expected to reduce the efficacy of selection and limit adaptive potential, repeated adaptation may nevertheless occur under shared environmental pressures. We further hypothesized that repeated adaptation could draw upon three alternative sources of adaptive variation: standing genetic variation inherited from a common ancestor, adaptive introgression through interspecific gene flow, or independent *de novo* mutations arising after lineage divergence. Distinguishing among these mechanisms enables explicit tests of how demographic history shapes the repeatability of adaptation and the evolutionary origins of adaptive genetic variation.

To evaluate these hypotheses, we conduct a comparative population genomic analysis of two closely related kiwifruit species, *Actinidia hemsleyana* (narrow‐ranged) and *Actinidia eriantha* (widespread), which differ markedly in demographic history and range size but occupy overlapping environments in subtropical China. This natural contrast provides a good system for examining how demographic limits, mutation load, and shared selective pressures interact to shape the genomic architecture of adaptation.

## RESULTS

### Demographic history predicts contrasting diversity and inbreeding

To test whether contrasting geographic ranges translate into divergent genomic consequences, we first characterized population structure, genetic diversity, and inbreeding in sympatric populations of narrow‐ranged *A. hemsleyana* and widespread *A. eriantha* (Table S2). Consistent with their distinct demographic histories, the two species formed well‐separated genetic clusters, confirming long‐term divergence despite local sympatry (Figure 1c; Figures S1 and S2).

**Figure 1:**
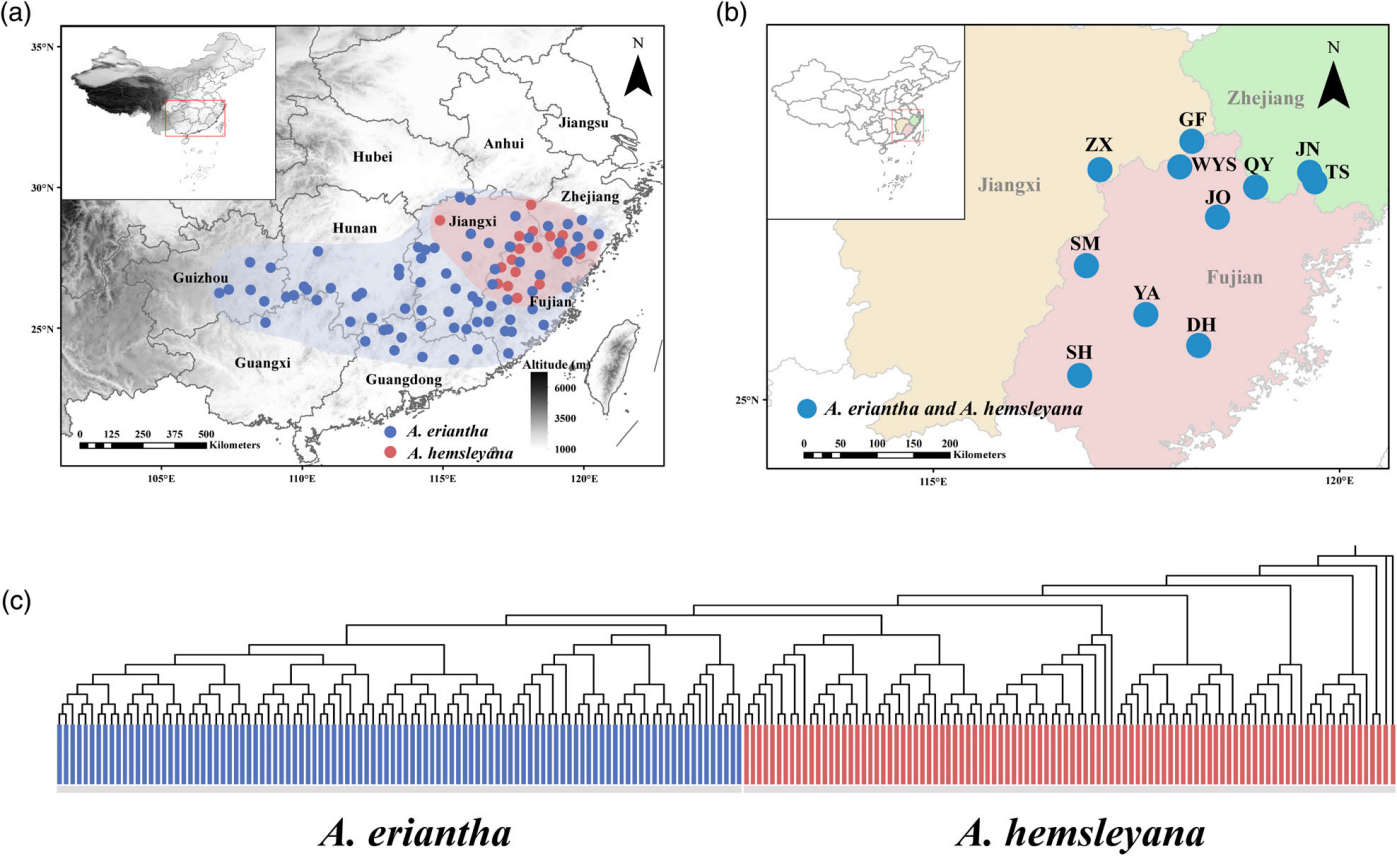
Geographic distribution, sampling map and population genetic structure for two *Actinidia* species. (a) Geographic distribution map of *Actinidia eriantha* and *Actinidia hemsleyana*. (b) Sampling locations of *A*. *eriantha* and *A*. *hemsleyana* used in this study. (c) Maximum‐likelihood phylogenetic tree with the best‐fit model of TVM + F + R7. Population genetic structure (*K* = 2) of 205 individuals; each individual is represented by a vertical bar.

As predicted under stronger genetic drift, the narrow‐ranged *A. hemsleyana* exhibited lower genome‐wide diversity and effective population sizes than the widespread *A. eriantha* (*θ*_*π*_ = 2.54 × 10^−3^ versus 3.76 × 10^−3^; *θ*_W_ = 1.43 × 10^−3^ versus 2.35 × 10^−3^; *N*_e_ = 27 908 versus 45 863) (Figure 2c,d; Table 1). The consistently higher nucleotide diversity (*θ*_*π*_) and Watterson's estimator (*θ*_W_) observed in *A. eriantha* compared to *A. hemsleyana* across multiple independent 100‐kb sliding windows indicated that the relative differences in genetic diversity between the two species were robust and unlikely to be significantly influenced by reference genome bias. This erosion of diversity was accompanied by marked reduced individual heterozygosity and elevated inbreeding, with *A. hemsleyana* showing nearly twofold higher genomic inbreeding coefficients (*F*_ROH_) (Figure 2a; Table S5). Across individuals, heterozygosity declined with increasing inbreeding, especially in the narrow‐ranged *A. hemsleyana* (Figure 2b), indicating that demographic contraction has reshaped genome‐wide variation in the range‐restricted species.

**Figure 2:**
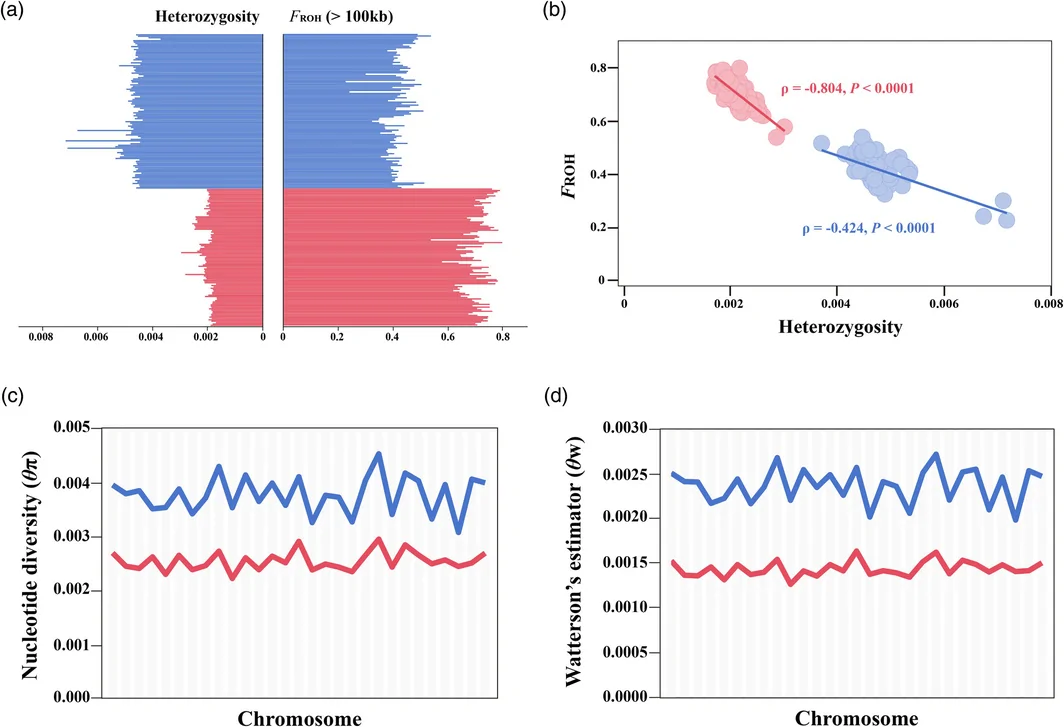
Genetic diversity and inbreeding. (a) Heterozygosity and *F*_ROH_ for each individual. Each individual is represented by a horizontal bar. ROH, runs of homozygosity. (b) Relationship between heterozygosity and *F*_ROH_ for each species. (c) Nucleotide diversity (*θ*_*π*_) averaged across each chromosome. (d) Watterson's estimator (*θ*_W_) averaged across each chromosome. The light gray bars of the *x*‐axis represent each chromosome. *Actinidia eriantha* and *Actinidia hemsleyana* are marked in blue and red, respectively.

**Table 1:** Genetic diversity statistics and effective population sizes at the species and population levels

		*Actinidia hemsleyana*					*Actinidia eriantha*				
		SNP	Individuals	*θ*_W_ (×10^−3^)	*θ*_*π*_ (×10^−3^)	*N*_e_	SNP	Individuals	*θ*_W_ (×10^−3^)	*θ*_*π*_ (×10^−3^)	*N*_e_
Species level		5 298 655	100	1.43	2.54	27 908 [CI: 27 799–28 014]	8 791 151	105	2.35	3.76	45 863 [CI: 45 765–46 099]
Population level	JN (Jingning, Zhejiang)	3 044 778	10	1.36	1.79	26 542 [CI: 26 414–26 662]	6 441 875	10	2.88	3.35	56 206 [CI: 55 990–56 415]
	TS (Taishun, Zhejiang)	3 077 692	10	1.37	1.80	26 737 [CI: 26 701–26 951]	6 483 442	10	2.90	3.38	56 596 [CI: 56 342–56 771]
	QY (Qingyuan, Zhejiang)	3 739 057	10	1.67	2.15	32 592 [CI: 32 462–32 739]	6 715 502	10	3.00	3.43	58 548 [CI: 58 344–58 793]
	WYS (Wuyishan, Fujian)	3 136 515	10	1.40	1.82	27 322 [CI: 27 212–27 469]	6 603 888	10	2.95	3.36	57 572 [CI: 57 382–57 829]
	JO (Jianou, Fujian)	3 231 205	9	1.49	1.91	29 079 [CI: 28 919–29 187]	6 160 459	9	2.84	3.24	55 425 [CI: 55 219–55 644]
	SM (Sanming, Fujian)	3 304 581	10	1.48	1.95	28 884 [CI: 28 688–28 933]	5 789 238	10	2.59	3.01	50 546 [CI: 50 286–50 699]
	YA (Yongan, Fujian)	3 404 828	10	1.52	1.97	29 664 [CI: 29 541–29 811]	6 229 356	10	2.78	3.22	54 254 [CI: 54 116–54 544]
	DH (Dehua, Fujian)	2 380 802	4	1.46	1.71	28 493 [CI: 28 247–28 538]	5 127 297	6	2.69	3.03	52 498 [CI: 52 305–52 749]
	SH (Shanghang, Fujian)	3 116 675	10	1.39	1.83	27 127 [CI: 27 036–27 296]	5 821 314	10	2.60	3.00	50 742 [CI: 50 556–50 977]
	GF (Guangfeng, Jiangxi)	3 086 592	10	1.38	1.81	26 932 [CI: 26 778–27 024]	6 443 853	10	2.88	3.34	56 206 [CI: 55 999–56 437]
	ZX (Zixi, Jiangxi)	2 568 064	7	1.28	1.61	24 980 [CI: 24 846–25 086]	5 550 642	10	2.48	2.92	48 400 [CI: 48 217–48 620]

Abbreviations: *θ*_W_, Watterson's estimator; *θ*_*π*_, nucleotide diversity; *N*_e_, effective population size.

### Demographic erosion translates into asymmetric mutation load

Reduced genetic diversity and elevated inbreeding are expected to weaken purifying selection, allowing deleterious mutations to accumulate. To evaluate whether this demographic contrast has translated into differential mutation load, we compared the accumulation and purging of putatively deleterious mutations between *A. hemsleyana* and *A. eriantha* using single‐nucleotide polymorphisms (SNPs) from protein‐coding genes (Table S6). Among these SNPs, 4196 in *A. hemsleyana* and 4230 in *A. eriantha* were predicted to be mildly deleterious based on Grantham scores (≥150), respectively; 3039 in *A. hemsleyana* and 2957 SNPs in *A. eriantha* were identified as strongly deleterious (loss‐of‐function, LoF) by SnpEff, respectively; and 565 in *A. hemsleyana* and 602 in *A. eriantha* were predicted to be deleterious by SIFT scores (<0.05), respectively. Based on the deleterious SNPs identified above, we compared derived deleterious variants between the two kiwifruit species. As expected, *A. hemsleyana* carried a higher proportion of deleterious alleles than *A. eriantha* (Figure 3; Table S7). We further assessed the relative mutation load between the two species and purging of different deleterious mutations using a standardized *R*_X/Y_ method. These analyses revealed an excess of strongly deleterious variants in *A. hemsleyana*, whereas mildly deleterious variants were not enriched and showed patterns consistent with partial purging under long‐term inbreeding relative to *A. eriantha* (Tables S8 and S9; Figure S3).

**Figure 3:**
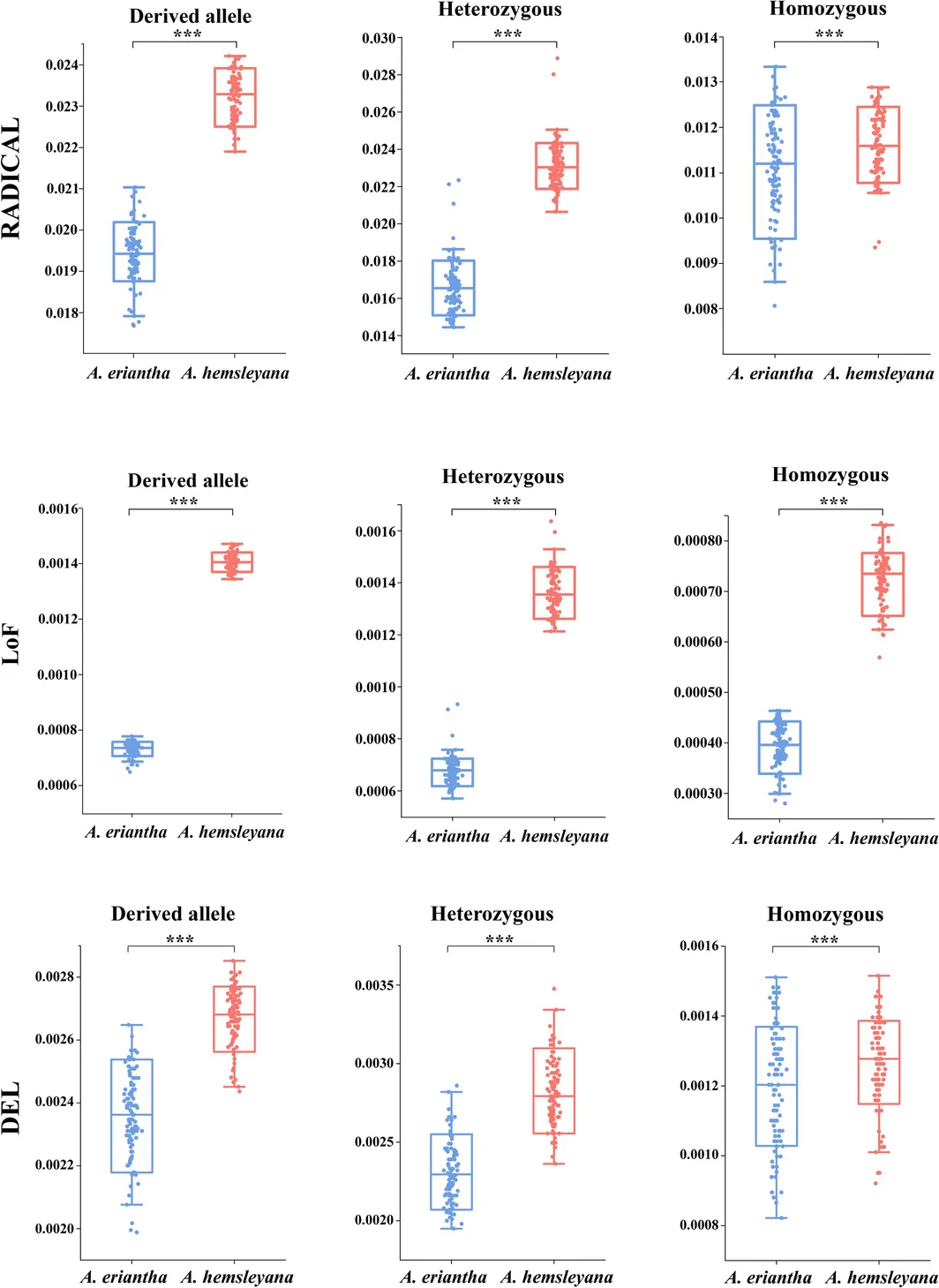
Accumulation of deleterious mutations in the two *Actinidia* species. Proportion of derived alleles, heterozygous genotypes, and homozygous derived genotypes in RADICAL loci (deleterious mutations predicted by Grantham scores), LoF loci (loss‐of‐function mutations predicted by SnpEff), and DEL loci (deleterious mutations predicted by SIFT scores) for *Actinidia eriantha* and *Actinidia hemsleyana*. ***, *P* < 0.001.

### Repeated adaptation despite demographic constraint

Despite pronounced differences in demographic history and mutation load, sympatric populations of the two species experience highly similar climatic environments. We therefore asked whether shared selective pressures have driven repeated genetic adaptation across species. Gradient forest (GF) and genotype–environment association (GEA) analyses consistently identified precipitation and solar radiation as dominant selective axes in both species (Figure 4a; Figures S5–S7).

**Figure 4:**
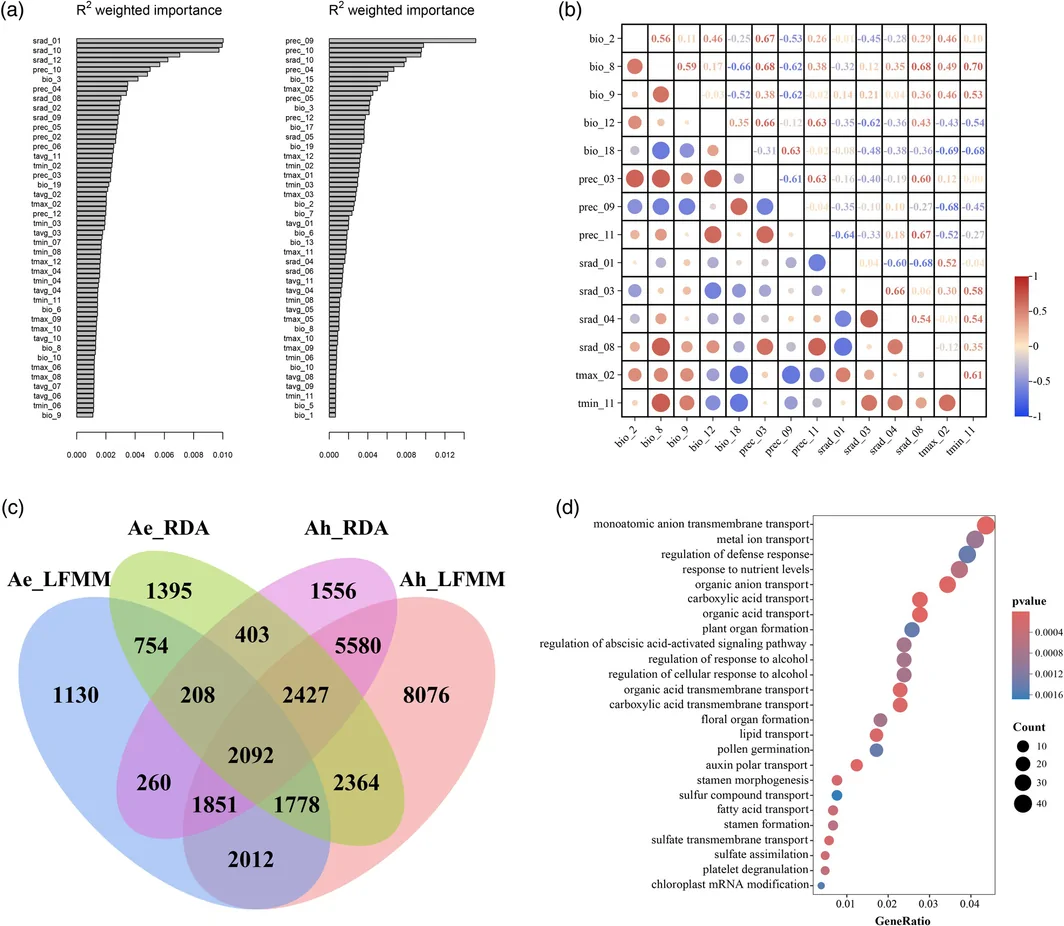
Genotype–environment associations (GEAs) performed separately in sympatric populations of *Actinidia eriantha* and *Actinidia hemsleyana*. (a) Importance ranking of environmental variables. Relative importance of all environmental variables based on gradient forest (GF) analysis of 50 000 randomly selected single‐nucleotide polymorphisms (SNPs) from *A. eriantha* (left) and *A. hemsleyana* (right). (b) Pairwise correlation coefficients between independent environmental variables. 14 independent environmental variables selected based on correlation coefficients (|*r*| < 0.7) for GEA analysis. (c) Venn diagram of candidate genes identified by two GEA approaches (latent factor mixed model [LFMM] and redundancy analysis [RDA]) in *A. eriantha* (Ae) and *A. hemsleyana* (Ah). (d) Gene Ontology (GO) enrichment of shared 2092 candidate genes associated with environmental factors between *A. eriantha* and *A. hemsleyana*.

Although environment‐associated SNPs were more diffusely distributed in *A. hemsleyana*, whereas *A. eriantha* showed more pronounced genomic clustering (Figure S9), both species shared a substantial set of adaptive candidates. Through functional annotation of the identified adaptive SNPs, a total of 2092 genes were associated with climatic variables in both species, revealing extensive gene reuse during adaptation to shared environments (Figure 4c). These genes were enriched for functions related to stress responses, hormone signaling, and reproductive development (Figure 4d; Table S10), indicating repeated involvement of similar biological pathways despite contrasting demographic backgrounds. However, at the SNP level, only two adaptive SNPs were shared between the two species (Figure S6a), suggesting that repeated adaptation in this system occurs predominantly through the reuse of genes rather than the same nucleotide sites.

To further resolve the genomic basis of repeated adaptation, we next examined signatures of recent positive selection within populations. Using complementary selective sweep approaches (SweeD and OmegaPlus), we identified candidate sweep regions across all populations of *A. hemsleyana* and *A. eriantha* (Table S11). Selected regions were distributed across the genome, with chromosomes 23, 21, 25, and 11 showing a higher proportion of sweep signals (Figure 5c). Across species and population pairs, 458 genes were consistently located within overlapping sweep regions and were designated as candidate targets of selection (Figure 5a; Table S12). These genes were significantly enriched for functions associated with responses to biotic or external stimuli, defense responses, and tropism (*P* < 0.01; Figure 5b).

**Figure 5:**
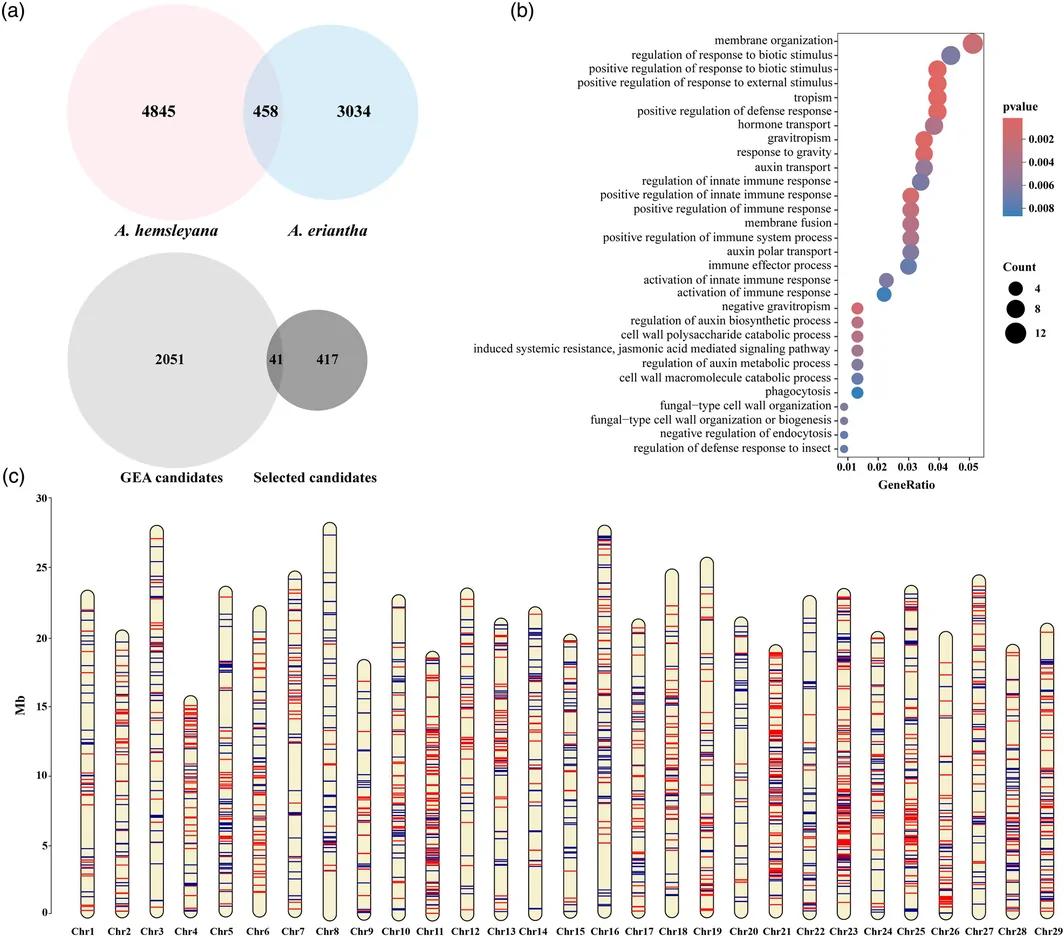
Signatures of selective sweep. (a) Above: Venn diagram showing the 458 positively selected genes shared between *Actinidia hemsleyana* and *Actinidia eriantha*. Below: Venn diagram showing the 41 genes overlapping between genotype–environment association (GEA) candidates (2092 genes shared between the two species, identified by genotype–environment associations) and positively selected candidates (458 genes shared between the two species, identified by selective sweeps). (b) Functional enrichment of 458 positively selected genes shared between the two kiwifruit species. (c) Genomic regions under positive selection identified in *A. hemsleyana* (red) and *A. eriantha* (blue).

By intersecting selective sweep candidates with environment‐associated genes, we derived a conservative set of 41 environmental adaptation candidates (Figure 5a; Table S13). These genes were significantly enriched for functions related to abscisic acid‐activated signaling, cellular response to stress, and regulation of innate immune responses (Figure S8), suggesting a shared genomic basis for repeated adaptation to climatic variation. It should be noted that the above adaptive candidates were inferred based on statistical association analyses, and whether they indeed contribute to adaptation in the two kiwifruit species requires further experimental validation.

### Repeated adaptation arises primarily from ancestral variation

Repeated adaptation can arise through distinct evolutionary mechanisms. We therefore asked whether the repeated adaptive signals observed in the two kiwifruit species reflect independent acquisition of adaptive variants or selection on shared ancestral variation. Coalescent‐based model comparisons (DMC method) strongly supported selection on ancestral standing genetic variation as the predominant source of repeated adaptation, accounting for nearly 90% of the candidate genes (Table S14). Models involving migration explained a small fraction of cases (9.8%), whereas independent *de novo* mutations were rare (2.4%). These results indicate that repeated adaptation between *A. hemsleyana* and *A. eriantha* has been achieved primarily through reuse of pre‐existing variation, rather than novel adaptive variants arising independently, suggesting the importance of ancestral genetic reservoirs in facilitating adaptation under demographic constraint.

## DISCUSSION

### Demographic limits shape genome‐wide diversity and inbreeding

Population genetic theory predicts that species with narrower geographic ranges and smaller effective population sizes should experience stronger genetic drift and higher inbreeding, resulting in reduced genetic diversity than their widespread relatives (Bortoluzzi et al., 2020; Eckert et al., 2008; Sheth et al., 2020). Although this expectation is well supported by population‐level studies and work on threatened taxa, genome‐wide tests in common species that differ primarily in range size remain relatively limited (Ma et al., 2021; Peng et al., 2025; Xie et al., 2025; Yang et al., 2024).

By comparing two closely related kiwifruit species sampled from sympatric regions, our study provides a clear genomic illustration of how demographic limits translate into contrasting evolutionary dynamics. The narrow‐ranged *A. hemsleyana* exhibited markedly reduced genetic diversity and elevated inbreeding relative to the widespread *A. eriantha*, consistent with its smaller effective population size and long‐term demographic decline (Yu et al., 2023). Similar contrasts between narrowly distributed and widespread congeners have been reported in other plant systems, including *Rhododendron*, *Quercus*, and *Dyckia* (Aguiar‐Melo et al., 2025; Kamiya et al., 2022; Zhu et al., 2025), suggesting that this pattern is not idiosyncratic to kiwifruit. Importantly, because both species were sampled from overlapping environments, the observed genomic differences are unlikely to reflect local ecological variation and instead underscore the dominant role of demographic history. Together, these results reinforce the view that geographic range size and long‐term population size impose pervasive constraints on genome‐wide variation, even among geographically proximate and ecologically similar species.

### Mutation load reflects reduced efficacy of purifying selection under demographic constraint

Reduced genetic diversity and elevated inbreeding are expected to weaken purifying selection and promote the accumulation of deleterious mutations (Bortoluzzi et al., 2020). In line with this expectation, the narrow‐ranged *A*. *hemsleyana*, which exhibited reduced genetic diversity and elevated inbreeding, carried a significantly higher burden of strongly deleterious variants than the widespread *A. eriantha*. This pattern is consistent with reduced efficacy of purifying selection under long‐term demographic contraction and mirrors observations from other systems experiencing population decline (Glémin, 2003; Liu et al., 2022; Welch et al., 2008; Yang et al., 2018). For example, excess LoF mutations have been reported in the narrowly distributed *Oreocharis esquirolii* relative to its widespread congener *Oreocharis maximowiczii* (Peng et al., 2025), as well as in the declining pheasant *Crossoptilon mantchuricum* (Wang et al., 2021), highlighting a common genomic consequence of sustained demographic limitation.

Theoretical work further predicts that the fate of deleterious mutations depends on their dominance and selection coefficients, with most deleterious alleles being at least partially recessive (Peischl & Excoffier, 2015; Yi et al., 2024). Increased homozygosity under inbreeding can therefore expose weakly deleterious alleles to selection, potentially leading to their partial removal, even as strongly deleterious variants continue to accumulate through drift. In line with this framework, we observed that *A. hemsleyana* carried a significantly higher burden of both strongly deleterious (LoF) and mildly deleterious variants, across total, heterozygous, and homozygous classes, as well as across realized and masked mutation load. Together, these results indicate that demographic constraints in *A. hemsleyana* have increased the burden of deleterious variation.

As a narrowly distributed species with limited geographic and genetic buffers, *A. hemsleyana* is more vulnerable to habitat loss, stochastic events, and future climate change. The reduced genetic diversity and elevated mutation load observed in this species further compound concerns about its future adaptive potential under climate change. From the conservation point of view, future efforts should take artificial crossing strategies to reduce risks of inbreeding depression and preclude the continuous decrease of genetic diversity. Although the numerous identified numerous candidate loci putatively involved in local adaptation that may offer some resilience to climate change, the extent to which these adaptive variants can withstand future climate changes remains uncertain, and therefore, further genomic vulnerability assessments will be essential to clarify its capacity for future adaptation and guide conservation prioritization.

### Repeated adaptation under contrasting demographic backgrounds

Despite pronounced differences in genetic diversity, inbreeding, and mutation load, sympatric populations of *A. hemsleyana* and *A. eriantha* experience highly similar climatic environments. This contrast raises a key evolutionary question: can adaptation remain repeatable under strong demographic constraint? Our results indicate that it can. GEA and selective sweep analyses revealed substantial overlap in adaptive targets between the two species, indicating repeated genetic adaptation to shared climatic gradients despite contrasting demographic histories.

Repeated adaptation, where independently evolving lineages respond to similar selective pressures in comparable ways, provides an important framework for assessing the predictability of evolution (Chaturvedi et al., 2022; Holliday et al., 2016; Lobkovsky & Koonin, 2012; Pool et al., 2017; van Boheemen & Hodgins, 2020; Yeaman et al., 2016). Such repeatability is often inferred from gene reuse, whereby selection repeatedly targets the same genes across lineages exposed to similar environments. Given the sympatric distribution of *A. eriantha* and *A. hemsleyana* in eastern China, we hypothesized that shared selective pressures could promote repeated adaptation through reuse of common genetic targets. Consistent with this expectation, GEA analyses identified 2092 shared candidate genes involved in local adaptation in both species, revealing extensive reuse of genes during adaptation to shared environments.

While both species exhibited evidence of gene reuse, the genomic architecture of adaptation differed between them. The narrow‐ranged *A. hemsleyana* harbored a substantially larger number of environment‐associated genes than the widespread *A. eriantha* (11 950 versus 4832), whereas a higher proportion of adaptive candidates in *A. eriantha* were shared between species (43.3 versus 17.5%). This asymmetry aligns with theoretical expectations for how gene flow shapes adaptive evolution. In widespread species experiencing higher gene flow, adaptation is often driven by a smaller number of genes with larger effects, whereas populations with restricted gene flow and smaller effective population sizes may respond through more polygenic shifts involving many genes of smaller effect (Savolainen et al., 2013). These results suggest that demographic context influences not whether repeated adaptation occurs, but how it is genetically realized.

The environmental drivers underlying this repeated adaptation were consistent between species. Notably, precipitation and solar radiation emerged as the dominant selective axes driving repeated adaptation in both kiwifruit species. This pattern is consistent with our previous large‐scale study demonstrating that these climatic variables are primary drivers of local adaptation in *A. eriantha* across its broad geographic range (Jiang et al., 2025). Independent physiological and ecological studies further underscore the central role of water availability and light conditions in shaping kiwifruit growth, stress tolerance, and reproductive performance (Guo et al., 2022; Jiang et al., 2022; Xiloyannis et al., 2023). Given the warm, humid monsoon climate of the southeastern coastal region and the tendency of kiwifruit to occupy riparian habitats and sun‐exposed forest gaps, repeated selection related to water availability and radiation is expected. Several of the 41 environmental adaptation candidates shared by both species encode genes previously implicated in responses to abiotic stress, including calcium‐dependent protein kinases, protein phosphatase 2Cs, and MYB transcription factors, which are involved in drought tolerance and oxidative stress responses (Bhaskara et al., 2017; Cheng et al., 2022; Ma et al., 2023; Wu et al., 2024). The repeated association of these genes with key climatic variables suggests that adaptation to shared environments has involved similar molecular pathways despite contrasting demographic backgrounds. Nonetheless, the precise functional mechanisms by which these genes influence environmental adaptation in kiwifruit require further experimental validation. Because candidate adaptive genes were identified through statistical GEA and selective sweep analyses, their functional roles remain hypotheses until experimentally validated.

### Ancestral standing variation and gene flow facilitate repeated adaptation

Repeated adaptation can arise through multiple evolutionary mechanisms, including the recruitment of ancestral standing variation, the independent origin of *de novo* mutations, as well as introgression via gene flow (Chaturvedi et al., 2025). Disentangling these mechanisms is central to understanding not only the predictability of evolution, but also how demographic history and evolutionary connectivity shape adaptive potential. In this study, we find that repeated adaptation to shared environments in *A. hemsleyana* and *A. eriantha* was driven predominantly by ancestral standing variation, with a secondary contribution from gene flow. The divergence time between *A. hemsleyana* and *A. eriantha* was estimated at approximately 9.1 Ma (95% confidence interval [CI]: 9.0–10.7 Ma), and the gene flow between them initiated at approximately 0.98 Ma (95% CI: 0.48–0.99 Ma) revealed in our previous study (Yu et al., 2023). Given that the timing of gene flow is substantially more recent than the species divergence time, this provides a temporal window during which ancestral polymorphisms could have persisted and been independently selected.

This pattern is consistent with theoretical expectations for closely related taxa with weak reproductive isolation and ongoing genetic exchange. Previous work has shown that *A. hemsleyana* and *A. eriantha* frequently hybridize under natural conditions, facilitating gene flow between species (Yu et al., 2023). Such connectivity increases the likelihood that adaptive alleles are shared across species, either through inheritance from a common ancestor or via introgression. Because these alleles are already segregating at appreciable frequencies, they can be rapidly recruited by selection when environmental conditions are shared.

Standing genetic variation is broadly expected to play a disproportionate role in repeated adaptation compared to *de novo* mutation. Pre‐existing alleles are immediately available to selection and may have already passed a degree of selective filtering in ancestral populations, reducing their deleterious effects relative to newly arising mutations (Thompson et al., 2019; Bohutínská & Peichel, 2024; Lai et al., 2024; Chaturvedi et al., 2025). As a result, adaptation from standing variation is often faster and more repeatable, particularly under environmental regimes that recur across space or time.

Empirical evidence for this mechanism is accumulating across diverse systems. For example, parallel serpentine adaptation in autopolyploid *Arabidopsis arenosa* is dominated by repeated recruitment of shared ancestral alleles (Konečná et al., 2021). Similarly, intercontinental genomic parallelism across multiple freshwater radiations of the three‐spined stickleback is largely founded on standing genetic variation (Magalhaes et al., 2021). In tropical butterflies, both standing variation and gene flow have been shown to facilitate repeated genetic adaptation to altitude across lineages (Montejo‐Kovacevich et al., 2022). Together with studies in plants and animals spanning a wide range of ecological contexts (Blanco‐Pastor et al., 2021; Bohutínská et al., 2021; Burke et al., 2014; Jacobs et al., 2020; Moran et al., 2023), these findings point to a general role for shared genetic variation in enabling repeatable adaptive responses.

In line with this broader framework, our results indicate that repeated adaptation in kiwifruit is not driven by widespread independent mutation, but instead reflects the selective reuse of pre‐existing genetic variation maintained through shared ancestry and ongoing gene flow. This mechanism provides a plausible explanation for how repeated adaptation to similar environments can persist even under asymmetric demographic constraints and elevated mutation load, reinforcing the view that demographic limitation shapes but does not eliminate the pathways through which adaptation proceeds.

## CONCLUSION

By contrasting two closely related kiwifruit species with divergent demographic histories but overlapping environments, this study reveals how demographic constraints shape evolutionary potential without eliminating adaptive repeatability. Consistent with population genetic theory, the narrow‐ranged *A. hemsleyana* shows reduced genetic diversity, elevated inbreeding, and a heavier burden of deleterious mutations relative to the widespread *A. eriantha*, reflecting long‐term differences in effective population size and the efficacy of purifying selection. Despite these constraints, both species exhibit repeated genomic responses to shared climatic gradients, with adaptation involving similar genes and pathways associated with precipitation and solar radiation. Demographic limitation therefore alters the genomic architecture of adaptation rather than preventing adaptation altogether. Our results further indicate that repeated adaptation is driven primarily by ancestral standing variation, with additional contributions from gene flow, highlighting the importance of shared ancestry and evolutionary connectivity. Together, these findings show how drift, mutation load, and shared variation interact to shape adaptive outcomes under demographic constraint. However, our findings are only based on a single species pair, and future studies should incorporate additional species pairs—particularly those spanning a broader range of demographic contrasts, to determine whether the patterns observed here are broadly generalizable.

## MATERIALS AND METHODS

### Target species

*Actinidia* Lindley (Actinidiaceae), a dioecious genus (i.e., male and female flowers on separate plants) with significant economic value, comprises 54 species and 21 varieties (Huang et al., 2013). The genus is widely distributed across East Asia, from tropical to low‐temperate zones. Most species are primarily distributed in the mountainous areas in China, where they show high morphological diversity and adaptability to diverse ecological conditions (Huang et al., 2013; Liu, Li, Zhang, et al., 2017; Liu, Li, Zhou, et al., 2017). In this study, we focused on two sympatric *Actinidia* species with contrasting distribution ranges, *A. eriantha* and *A*. *hemsleyana*. These two species share highly similar life histories, including mating system, dispersal mode and habitat type. As an important taxon for introgressive breeding of new kiwifruit cultivars, *A. eriantha* is widely distributed across subtropical China (100–1500 m elevation), including Zhejiang, Fujian, Jiangxi, Hunan, Guangdong, Guangxi, and Guizhou (Huang et al., 2013; Testolin et al., 2016). In contrast, *A*. *hemsleyana* has a narrower distribution in eastern China where both species usually co‐occur below 1000 m elevation (Figure 1a). In addition, both species shared with the same pollinators and flowering time in our filed observation.

### Plant materials acquisition and resequencing

Fresh young leaves of 205 individuals were collected from 11 natural sympatric distribution areas of *A. hemsleyana* and *A. eriantha* in Zhejiang, Fujian, and Jiangxi provinces of China, where the geographic distributions of these two species overlap (Figure 1b; Table S1). Additionally, a population with 10 individuals of *Actinidia lanceolata* from the overlap region was collected as an outgroup. The fresh leaves of each individual were dried with silica gel for DNA extraction.

Genomic DNA was extracted following a modified cetyltrimethylammonium bromide protocol (Doyle & Doyle, 1990), and then fragmented randomly to 200–400 bp for library construction. Whole genome resequencing was carried out on a DNBSEQ platform with a target depth of 10×. Resequencing data were obtained for all individuals, with an average depth of 11.6× (Table S2).

### Read mapping, SNP calling, and filtering

Raw reads were filtered using fastp v.0.22.0 (Chen et al., 2018) to remove adapter and low‐quality sequences, generating about 7 Gb of clean data for each sample. Clean paired‐end reads were mapped to the reference genome of *A. eriantha* (Yao et al., 2022) using the MEM algorithm of BWA v.0.7.17 (Li & Durbin, 2009). To evaluate potential bias caused by reference genome choice for mapping reads, we compared mapping rates between the two species. The average mapping rate was 96.0% for *A. eriantha* and 95.1% for *A. hemsleyana*, respectively (Table S2), indicating that reference genome choice does not induce substantial alignment bias. The resulting aligned reads were sorted and converted into binary alignment map format using SAMtools v.1.13 (Danecek et al., 2021). Duplicate reads resulting from PCR amplification were then removed using MarkDuplicates tool from Picard v.2.24.0 (https://github.com/broadinstitute/picard) with default parameters.

Variant detection for each sample was performed using the HaplotypeCaller module in GATK v.4.2.0 (DePristo et al., 2011), followed by joint genotyping with the GenotypeGVCFs tool. High‐quality biallelic SNPs were selected by VariantFiltration with the following stringent filtering criteria: QD <2.0, MQ <40.0, FS >60.0, SOR >3.0, MQRankSum <−12.5, ReadPosRankSum <−8.0. To retain high‐confidence SNPs, further filtration was performed using VCFtools v.0.1.16 (Danecek et al., 2011) with the following parameters: ‐‐minQ 30, ‐‐maf 0.05, ‐‐max‐alleles 2, ‐‐min‐alleles 2, ‐‐max‐missing 0.9. Finally, a total of 13 248 734 high‐quality SNPs within and across species were retained after stringent filtering standards. To enable separate estimates of genetic diversity for each species, we generated a final set of 5 298 655 SNPs for *A. hemsleyana* and 8 791 151 SNPs for *A. eriantha*, respectively.

### Population structure analysis

To analyze population genetic structure, highly correlated SNPs were further filtered through linkage disequilibrium (LD)‐based SNP pruning using PLINK v.1.9.0 (Purcell et al., 2007). The genome was scanned using sliding windows of 50 SNPs with a step size of 10 SNPs; any SNP that showed a correlation coefficient (*r* ^2^) >0.2 with another SNP within the same window was excluded, resulting in 1 185 780 independent SNPs for subsequent analyses. In order to infer the phylogenetic relationship, we used ModelFinder (Kalyaanamoorthy et al., 2017) to evaluate 483 DNA substitution models, ultimately selecting ‘TVM + F + R7’ as the optimal model based on the Bayesian Information Criterion. The phylogenetic tree was then constructed through the maximum‐likelihood (ML) method in IQ‐TREE v.1.6.12 (Nguyen et al., 2015), employing 1000 bootstrap iterations. In addition, we used ADMIXTURE v.1.3.0 (Alexander et al., 2009) to analyze the population genetic structure among all samples, with the number of clusters (*K*) being set from 2 to 10. Lastly, PLINK software was used to perform principal component analysis (PCA).

### Genome‐wide diversity and inbreeding

We calculated polymorphism statistics at the population and species levels using PIXY v.1.0.0 (Korunes & Samuk, 2021) and VCFTOOLS v.0.1.16 along the genome in 100 kb non‐overlapping windows, including nucleotide diversity (*θ*_*π*_) and Watterson's estimator (*θ*_W_). Contemporary effective population size (*N*_e_) for each species and population was calculated using the formula: *N*_e_ = $\frac{\theta_{W}}{4\times \mu }$ (Wright, 1931), with a mutation rate (*μ*) of 1.83 × 10^−9^ per site per year and a generation time of 7 years (Yu et al., 2023). Both parameters were specifically estimated for *Actinidia* species (Yu et al., 2023), ensuring that the mutation rate and generation time used here are taxonomically matched to our study system. We also calculated 95% CIs for *N*_e_ by applying the 1000‐replicate bootstrap procedure. To minimize the influence of unequal sample sizes between the two species, we randomly subsampled individuals to equalize the number of individuals per species and reassessed all genetic diversity statistics. The results indicated that the minor difference in sample size has no impact on the main conclusions of this study (Table S15).

Genome‐wide heterozygosity, determined by the ratio of heterozygous sites to the total number of callable sites, was calculated for each individual using realSFS implemented in ANGSD (Korneliussen et al., 2014). Next, we identified ROH for each individual. To this end, we initially conducted LD pruning (similar to genetic structure analysis) and removed rare alleles (MAF <0.05) for all SNPs. We then used PLINK to scan the entire genome with sliding windows of 50 SNPs. A ROH candidate was defined as being longer than 100 kb and containing at least 50 SNPs. No heterozygotes and a maximum of five missing calls were allowed in each sliding window. We then calculated individual inbreeding coefficients (*F*_ROH_) (Kardos et al., 2015) by summing the proportion of the genome covered by ROHs (total length of ROHs/total length of genome assembly).

### Estimation of the genetic load

We used the proportion of derived deleterious mutations detected for each individual to estimate genetic load. To polarize variable sites into ancestral and derived states, we included the sequencing data of *A. lanceolata*. The probability of derived versus ancestral allelic state of each SNP locus was inferred using est‐sfs v.2.03, with a probability cutoff set at 0.95 (Keightley & Jackson, 2018). To mitigate bias from potential deleterious mutations in the reference genome, we retained only SNPs with confirmed ancestral states where the ancestral allele matched the reference genome. The effects of SNPs on protein‐coding gene sequences were then annotated and classified into synonymous, nonsynonymous, and putative LoF mutations using SnpEff v.5.0 (Cingolani et al., 2012). We further classified nonsynonymous mutations by calculating Grantham scores (Grantham, 1974), which quantify the extent of amino acid change. Following established thresholds, missense mutations with a Grantham score ≥150 were categorized as deleterious (RADICAL loci), while those <150 were classified as benign. To minimize method‐specific bias, we additionally performed deleterious mutation prediction using SIFT4G (Vaser et al., 2016), and SNPs with a SIFT score <0.05 were defined as deleterious (DEL loci).

The deleterious mutation load was calculated based on the following two indexes, as described by Feng et al. (2024). We first calculated the ratio of deleterious mutations in each category (LoF and deleterious) as: *p*_a_ = $\frac{N_{\text{derived alleles}}^{\text{class}}}{N_{\text{SNPs}}^{\text{total}}\times 2}$. Here, $N_{\text{derived alleles}}^{\text{class}}$ represents the number of derived alleles in each category of SNPs, while $N_{\text{SNPs}}^{\text{total}}$ is the total number of SNPs used for deleterious mutation annotation. Next, we calculated the proportion of heterozygous and homozygous derived genotypes in each category of deleterious mutation loci as: *p*_g_ = $\frac{N_{\text{genotype}}^{\text{class}}}{N_{\text{SNPs}}^{\text{total}}}$. The values of *p*_a_ and *p*_g_ were calculated for all samples and compared across species using Wilcoxon tests. To further investigate the differences in the purging of deleterious mutations between populations, we calculated the total expected likelihood value of derived alleles found in population X but not in a randomly selected genome from population Y, and vice versa (Do et al., 2015; Xue et al., 2015). The ratio of these two counts (*R*_X/Y_) was then calculated following the detailed formulas as described by Xie et al. (2021). The intergenic SNPs assumed to be neutral were used for standardization following Do et al. (2015). In a particular group of potential deleterious sites, an *R*_X/Y_ value >1 indicates that population X has a higher number of derived deleterious mutations compared to population Y. Conversely, an *R*_X/Y_ value <1 suggests that population X has fewer derived deleterious mutations, implying that it is more effective at purging these deleterious mutations than population Y. Finally, to assess whether deleterious mutations in small populations led to a reduction in fitness, we used homology‐based inferences to functionally annotate genes with fixed deleterious mutations in each population of *A. hemsleyana*.

### Identification of environment‐associated variants

A total of 60 environmental factors and 19 climatic factors were extracted from the WorldClim2 database (https://www.worldclim.org/data/worldclim21.html) and assigned into three basic categories: temperature, precipitation and solar radiation (see Table S3 for all variables). We first conduct GF analyses with R package ‘gradientForest’ (Ellis et al., 2012) to evaluate the primary environmental factors influencing genetic variation in *A. hemsleyana* and *A. eriantha* datasets, respectively. GF was run using 50 000 random SNPs selected from the whole dataset with 5 replications and the following parameters: ntree = 500, maxLevel = log2(0.368*n*)/2, correlation threshold = 0.5. After considering the ranked importance based on GF analyses and the correlations among all environmental variables, 14 variables with correlation coefficient |*r*| < 0.7 were retained for the GEA analyses to avoid issues due to multicollinearity, including 5 temperature variables (bio_2, bio_8, bio_9, tmax_02 and tmin_11), 5 precipitation variables (bio_12, bio_18, prec_03, prec_09, and prec_11), and 4 solar radiation indices (srad_01, srad_03, srad_04, and srad_08) (Figure 4b; Figure S4).

Population structure may affect the identification of adaptive SNPs. Two different approaches explicitly account for population structure were used to perform GEA analyses on the non‐missing datasets of *A. hemsleyana* and *A. eriantha* (Chang et al., 2025; Lasky et al., 2023). Firstly, a univariate latent factor mixed model (LFMM) was implemented in the R package LEA v.3.3.2 (Frichot & François, 2015) to investigate associations between allele frequencies and the 14 environmental variables. LFMM uses latent factors to model unobserved population structure, with the number of latent factors (*K*) determined by cross‐validation, which represents the optimal number of latent factors for capturing population structure. Genomic inflation factors (GIF) were used to calibrate the association value between the frequency of each locus and environmental variable. We then used the R package *qvalue* (https://github.com/StoreyLab/qvalue) to convert *P*‐values for all SNPs into *q*‐values, considering SNPs with *q*‐values <0.01 as significant environment‐associated variants, and the candidate SNPs were retained if they exhibited a correlation with at least one environmental variable. Secondly, variants showing a particularly strong relationship with multivariate environmental axes were identified using a redundancy analysis (RDA), which has been demonstrated as one of the most effective multivariate GEA methods while maintaining low false‐positive rates (Capblancq & Forester, 2021). RDA incorporates population structure as conditioning variables via PCA axes, ensuring that the identified outlier loci reflect environmental adaptation. We performed the RDA analyses using the R package *vegan* with the first three principal components as conditional variants and selected the first three significant RDA axes to identify candidate SNPs. Outliers associated with environment variables were defined as those SNPs with a standard deviation cutoff of 3. To minimize false positives, we defined the final candidate set as the intersection of SNPs that were identified as significantly associated with environmental variables by both LFMM and RDA analyses.

### Signatures of selective sweep

The genome regions under selection were identified by SweeD v.4.0.0 (Pavlidis et al., 2013) and OmegaPlus v.3.0.3 (Alachiotis et al., 2012). Both methods detect within‐population sweeps, and comparing within‐species sweeps at shared localities offers a more direct test of repeated adaptation to similar environmental pressures. The composite likelihood ratio (CLR) statistics in SweeD were calculated with default parameters with 2 kb intervals. OmegaPlus statistics (*ω*) were computed at 2 kb intervals, and a minimum window of 500 bp and a maximum window of 10 kb were specified to be used for computing LD values between SNPs. To reduce false‐positive outliers derived from demographic processes, we performed coalescent simulations to establish the cutoff values for the CLR and *ω* statistics, as described in Wei et al. (2023). Using the coalescent simulator SCRM (Staab et al., 2015), we generated 10 000 neutral datasets based on the demographic history of each population (Yu et al., 2023). For each simulated dataset, we calculated the maximum CLR and *ω* values using SweeD and OmegaPlus, respectively. The 95th percentile of the maximum‐value distribution across the 10 000 simulations served as the outlier threshold. As this simulation‐derived threshold was less stringent than the top 1% empirical threshold, we adopted the latter (top 1% of windows from both methods) as candidate selective regions for each species to ensure conservatism. Using the genomic coordinates, we then extracted only the overlap regions between the two methods, which are regarded as high confident selective sweep regions.

### The mechanism of repeated adaptation across species

A coalescent modeling approach was used to test for patterns consistent with alternative modes of repeated evolution in our candidate genes. The Distinguishing Modes of Convergence (DMC) method developed by Lee and Coop (2017) was implemented in the R package rdmc (Tittes, 2020). DMC uses population genetic data to (i) model neutral evolution (no selection) and several alternative modes of repeated evolution (Moran et al., 2023). (ii) A model of ancestral standing variation assumes that the adaptive allele was segregating in the ancestral population and then repeatedly selected in each population. (iii) A model of migration assumes an adaptive allele evolved in one species and then spread among other species via gene flow. (iv) A model of *de novo* mutation assumes that the adaptive allele evolved independently in different species. The scenarios (ii) and (iii) were considered collectively as ‘shared origin’ because both processes act on alleles of a single mutational origin, in contrast to scenario (iv).

For each of the candidate genes for repeated adaptation, we provided DMC with allele frequencies from the two sympatric populations and two allopatric populations from the two kiwifruit species plus 10 kb upstream and downstream of each gene. We specified sympatric populations of two species as the populations under selection for the genes analyzed. To allow DMC to contrast neutral and selected allele frequency covariances among populations, we extracted fourfold degenerate synonymous sites (4DTv) throughout the genome, resulting in a set of 14 389 neutral allele frequencies. DMC calculates the composite likelihood for each alternative model at each gene along specific genomic region through using a grid of parameter values. We specified an effective population size of 50 000 (which is close to the average effective population across both kiwifruit species) and a recombination rate of 5 cM Mb^−1^ (Liu, Li, Zhang, et al., 2017; Liu, Li, Zhou, et al., 2017). We used all possible combinations of values specified for the selection coefficient, standing time (number of generations before the onset of selection and before migration from the source population occurred), migration rates, and frequency of the standing variant. Specific parameter values used were presented in Table S4. To quantify the relative contributions of ancestral standing variation, migration, and independent *de novo* mutations to repeated adaptation, the DMC method computes the maximum composite likelihood for each model as a measure of how well the model explains the observed data. For each gene, the model yielding the highest likelihood is selected as the most likely evolutionary scenario.

### GO enrichment

To elucidate the biological significance of our candidate genes, we performed Gene Ontology (GO) enrichment analyses to detect statistically overrepresented functional categories. The protein sequences were aligned against the local eggNOG‐mapper database (Cantalapiedra et al., 2021) using MMseq2 with default parameters (Steinegger & Söding, 2017). AnnotationForge (https://bioconductor.org/packages/AnnotationForge) was then used to construct the GO database. We performed GO enrichment analysis using the R package *Clusterprofiler* (Wu et al., 2021).

## AUTHOR CONTRIBUTIONS

XY designed the research. JH, YS, and QJ collected samples and coordinated research activities. MQ analyzed the data and wrote initial drafts of the manuscript. XY and NW reviewed and edited the manuscript. All authors read and approved the final manuscript.

## CONFLICT OF INTEREST

The authors declare no conflicts of interest.

## Supporting information


**Figure S1.** Principal component analysis (PCA) for all *A*. *hemsleyana* and *A*. *eriantha* populations, showing the first two components (PC1 and PC2) and the percentage of variation explained by each component.
**Figure S2.** Population genetic structure for all individuals of *A*. *hemsleyana* and *A*. *eriantha*, each individual is represented by a vertical bar.
**Figure S3.** GO enrichment analysis of genes with loss‐of‐function (LoF) or mildly deleterious mutations in *A. hemsleyana*.
**Figure S4.** Correlation coefficients of 14 environmental factors are calculated using *pairs panels*.
**Figure S5.** Redundancy analysis showing the relationships between the independent environmental factors and population structure of (a) *A*. *hemsleyana* and (b) *A*. *eriantha*.
**Figure S6.** Outlier SNPs associated with 14 environmental variables.
**Figure S7.** Importance ranking of all environmental variables based on gradient forest (GF) analysis of 50 000 randomly selected SNPs from (a) *A*. *eriantha* and (b) *A. hemsleyana* (four replicates per species).
**Figure S8.** GO enrichment analysis of the 41 environmental adaptation candidates, which represent the overlap between the GEA candidates (shared genes identified through genotype‐environment association analyses) and selected candidates (shared genes identified through selective scans).
**Figure S9.** Genome‐wide distribution of adaptive SNPs associated with environments factors in (a) *A. eriantha* and (b) *A. hemsleyana*, respectively. Tracks from outermost to innermost are (a) Chr number, (b) gene density on the whole genome, (c) SNP density, (d) indels density, and the distribution of potentially climatic‐adaptive (e) SNPs and (f) indels identified by both LFMM and RDA across the genome.

**Table S1.** Sample information of *A. hemsleyana* and *A. eriantha* used in this study.
**Table S2.** Summary of genome resequencing data for 100 individuals of *A. hemsleyana*, 105 individuals of *A. eriantha*, and 10 individuals of outgroup used in this study.
**Table S3.** Details of the environmental factors extracted from the WorldClim database for each population included in this study.
**Table S4.** Parameter values specified to DMC.
**Table S5.** Heterozygosity and inbreeding coefficient (*F*ROH) for each individual.
**Table S6.** Number of SNPs located in coding regions and number of SNPs predicted to be deleterious.
**Table S7.** Mean proportion of derived alleles, heterozygous and homozygous derived genotypes in each class of deleterious mutations for *A. hemsleyana* and *A. eriantha* (distributions of these statistics are shown in Figure 3).
**Table S8.** *R*_X/Y_ statistics of *A. hemsleyana* and *A. eriantha* across mildly deleterious (DEL), loss‐of‐function (LoF), and neutral mutation categories.
**Table S9.** Annotation information of genes harboring loss‐of‐function (LoF) or mildly deleterious mutations in *A. hemsleyana*.
**Table S10.** Annotations for shared candidate genes associated with environmental factors, identified in both *A. hemsleyana* and *A. eriantha* through LFMM and RDA analyses.
**Table S11.** Summary of selective sweeps in *A. hemsleyana* and *A. eriantha* populations.
**Table S12.** Annotations for candidate genes under positive selection shared between *A. hemsleyana* and *A. eriantha*.
**Table S13.** Annotations for candidate genes identified by both genotype–environment association and selection scan analyses.
**Table S14.** The source of candidate genes overlapping both GEA (genotype–environment association) and positive selection candidates.
**Table S15.** Genetic diversity statistics at the species and population levels for *A. eriantha* randomly subsampled to equalize sample sizes with *A. hemsleyana*.

## Data Availability

The raw resequencing data of *Actinidia hemsleyana* and *Actinidia eriantha* produced in this study have been submitted to the Genome Sequence Archive (GSA) under BioProject accession number CRA038619.
