# Supplementary material for: Repeated genetic adaptation under demographic limits in kiwifruit
Source: Plant J. 2026 Oct 12;128(1):e71163. doi: 10.1111/tpj.71163 (PMC13655936; doi:10.1111/tpj.71163)
Supplement: Supplementary file 1 [file TPJ-128-0-s002.pdf]

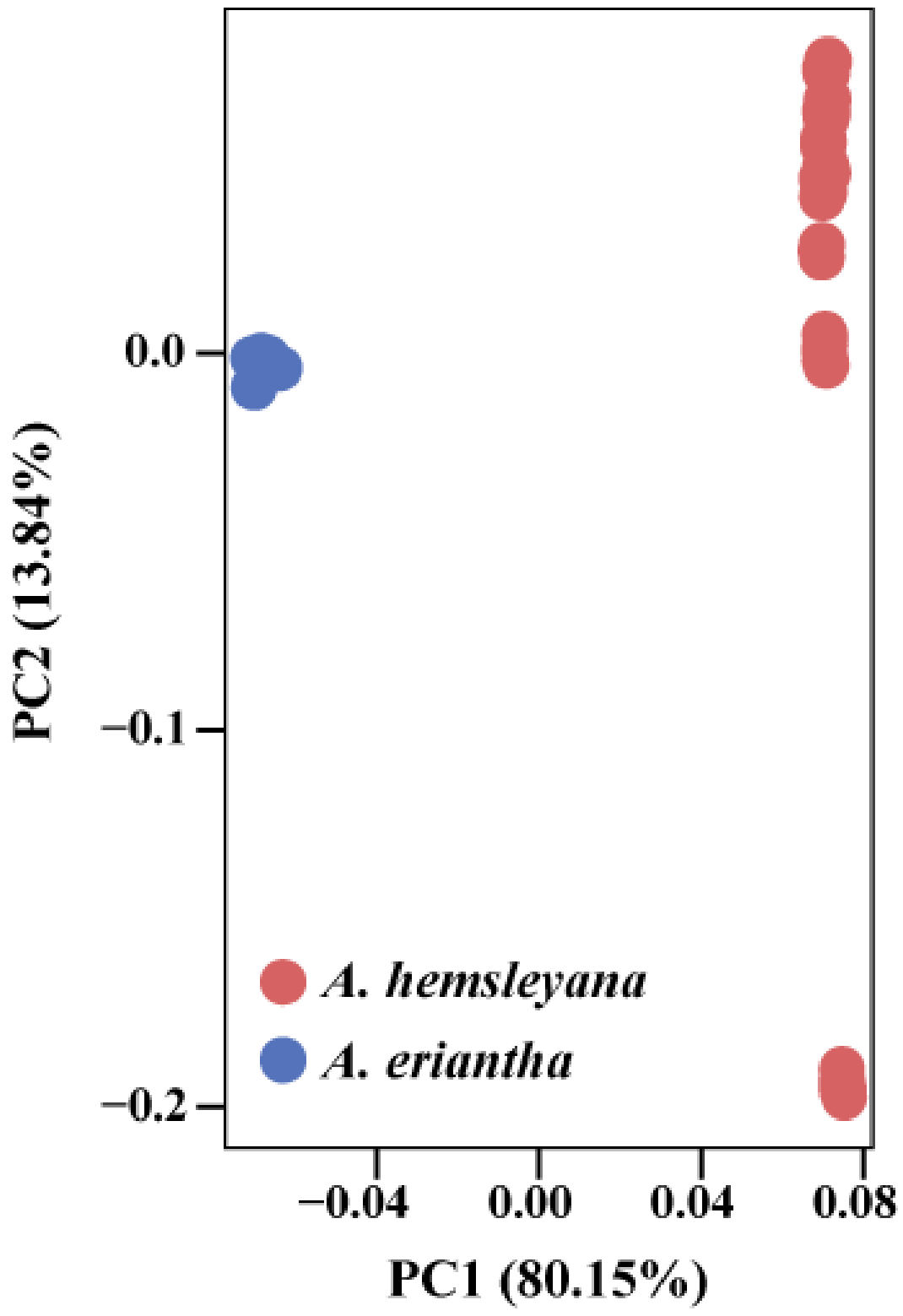

Fig. S1. Principal component analysis (PCA) for all *A. hemsleyana* and *A. eriantha* populations, showing the first two components (PC1 and PC2) and the percentage of variation explained by each component.

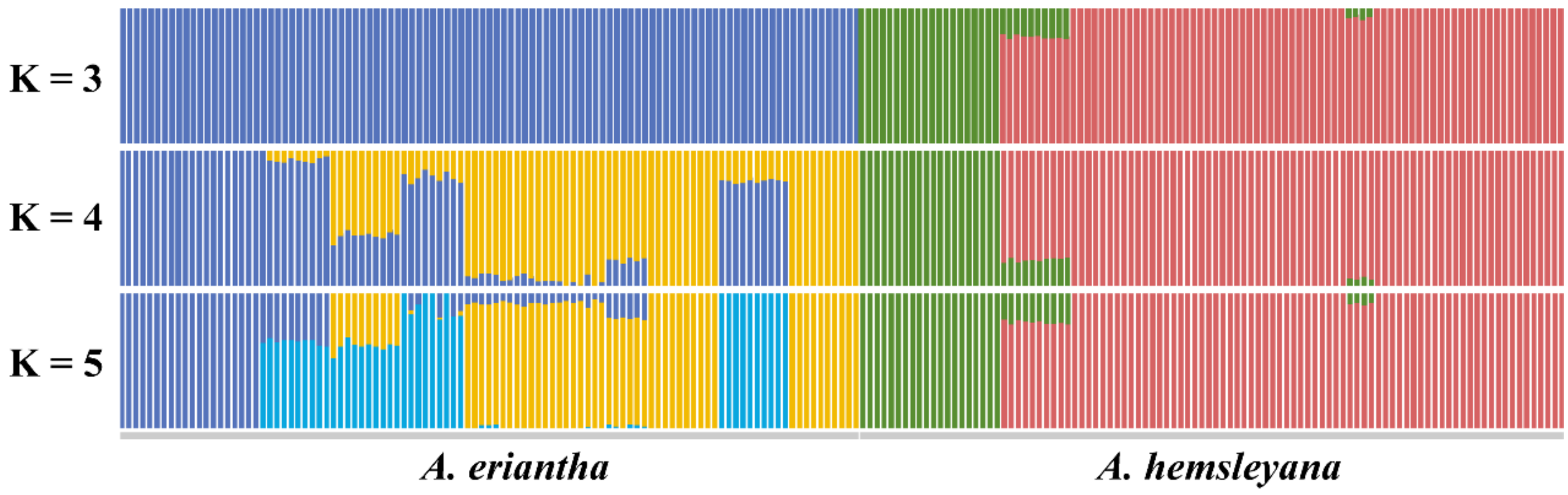

Fig. S2. Population genetic structure for all individuals of *A. hemsleyana* and *A. eriantha*, each individual is represented by a vertical bar.

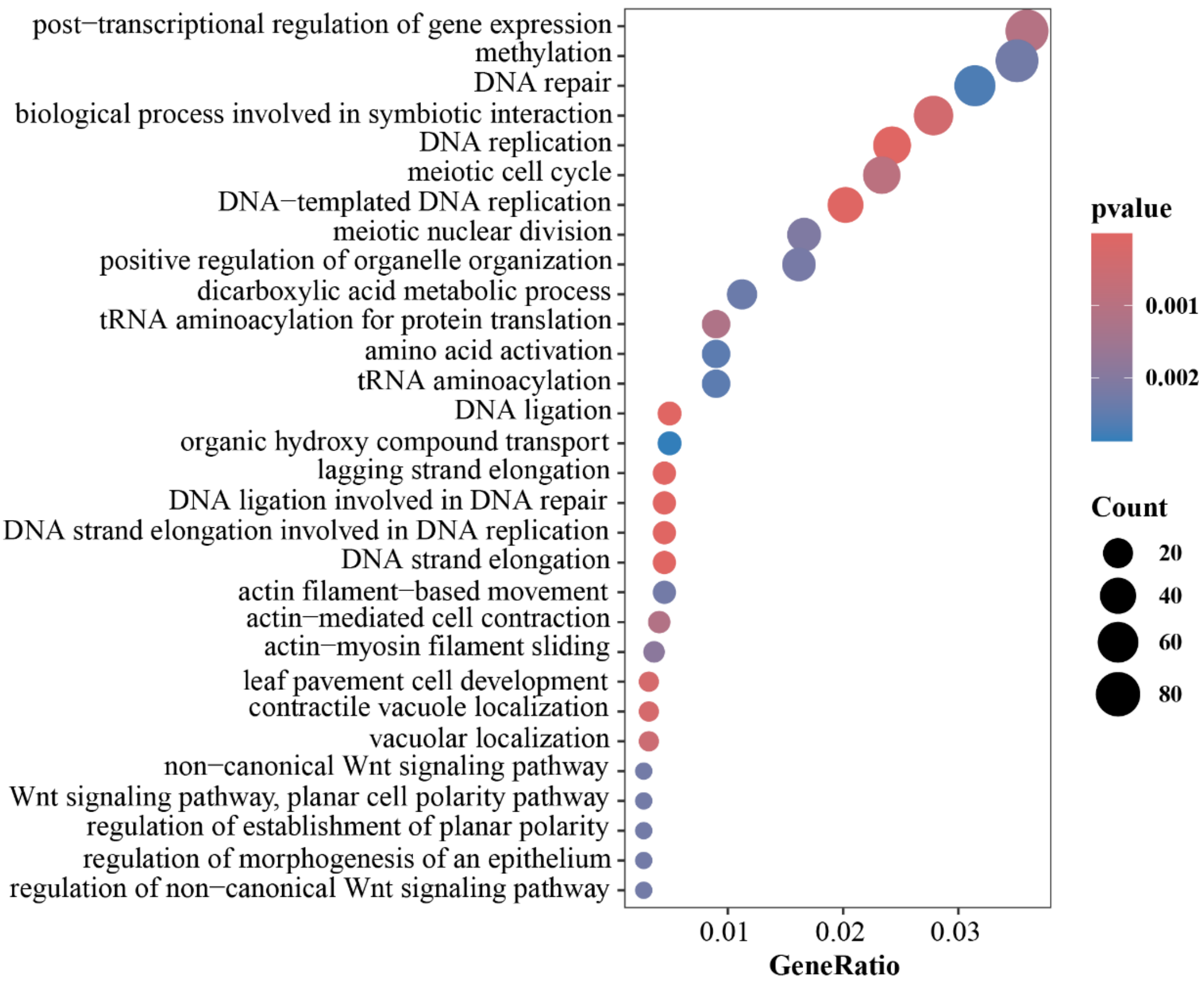

Fig. S3. GO enrichment analysis of genes with loss-of-function (LoF) or mildly deleterious mutations in *A. hemsleyana*.

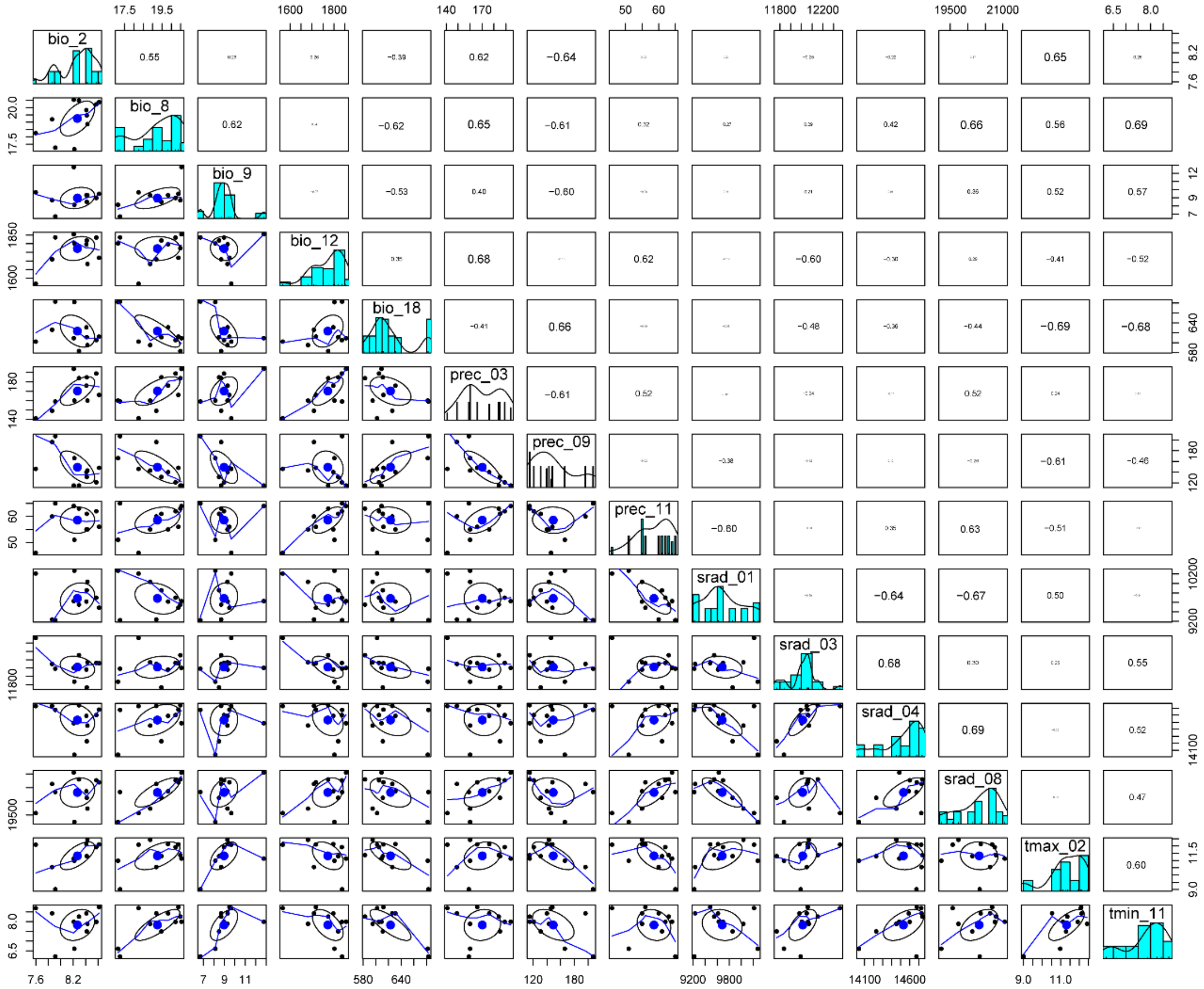

Fig. S4. Correlation coefficients of 14 environmental factors are calculated using *pairs. panels*. The values are showed in the upper right diagonal, with their size scaled to their |*r*|. The lower left shows scatter plots, while the diagonal shows histograms of the data.

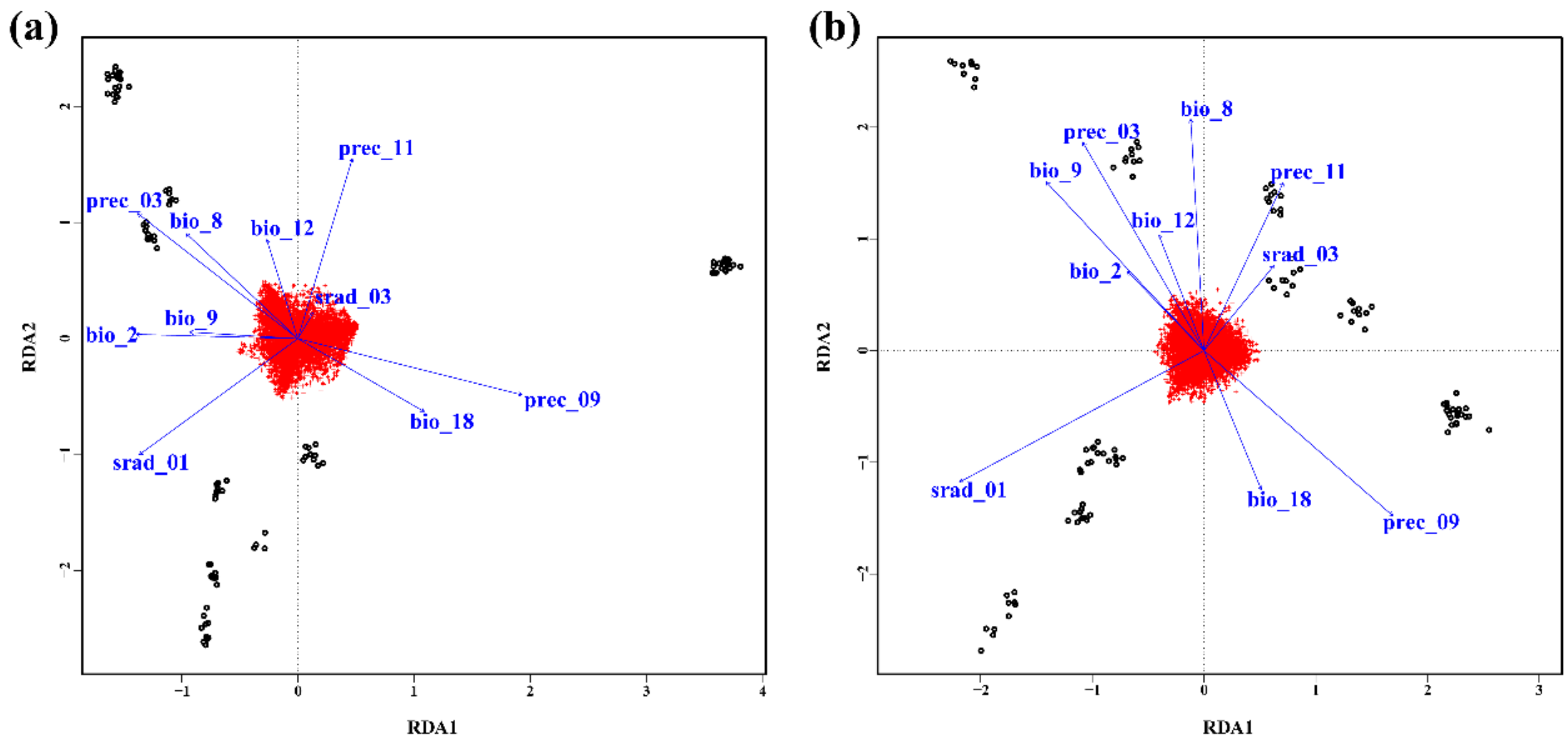

Fig. S5. Redundancy analysis showing the relationships between the independent environmental factors and population structure of (a) *A. hemsleyana* and (b) *A. eriantha*. Black circles are individuals. Small red points are SNPs. Blue vectors represent environmental variables.

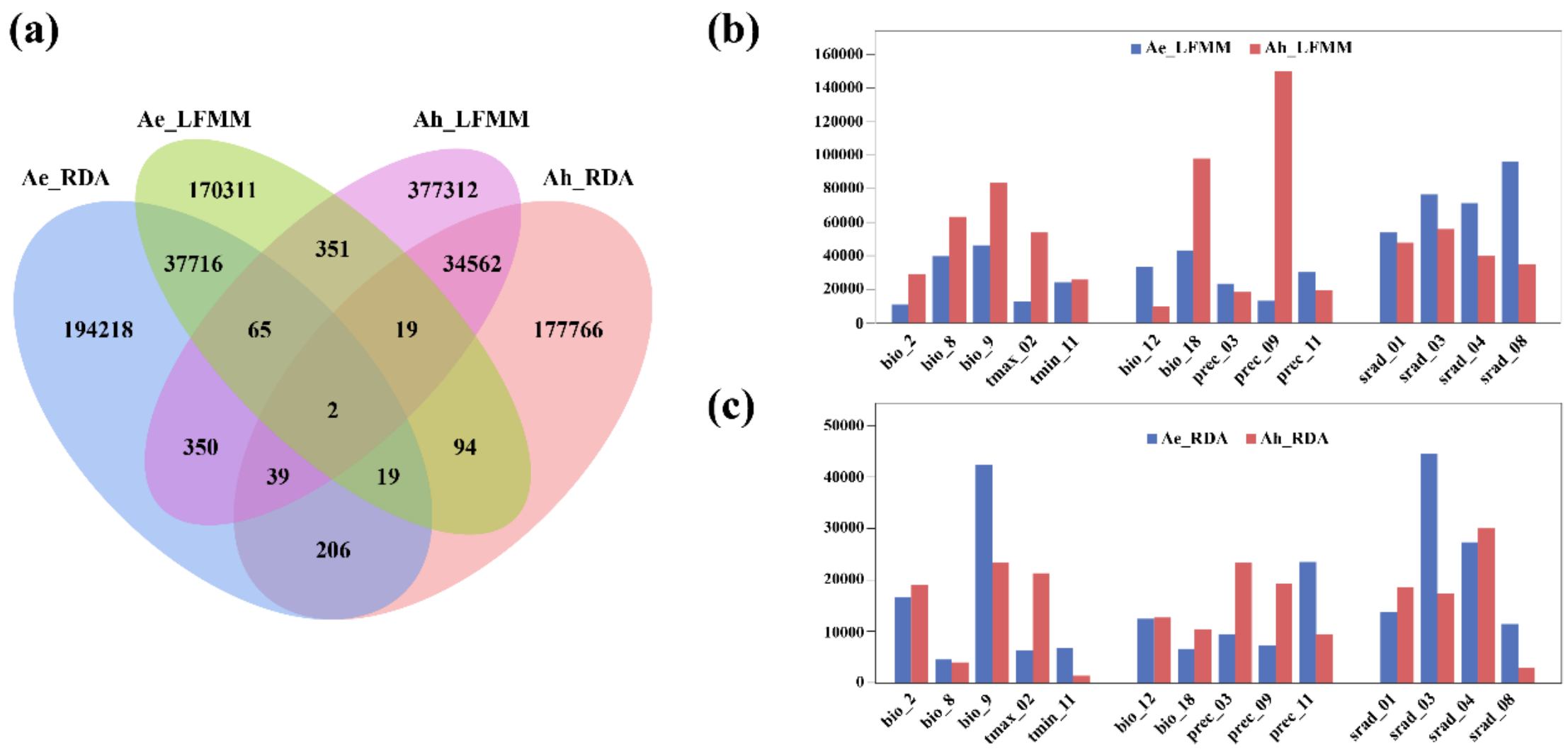

Fig. S6. Outlier SNPs associated with 14 environmental variables. (a) Venn diagram of adaptive SNPs identified in *A. hemsleyana* (Ah) and *A. eriantha* (Ae) through two GEA approaches (LFMM and RDA). The number of outlier SNPs associated with each environmental factor identified through (b) LFMM, and (c) RDA analyses.

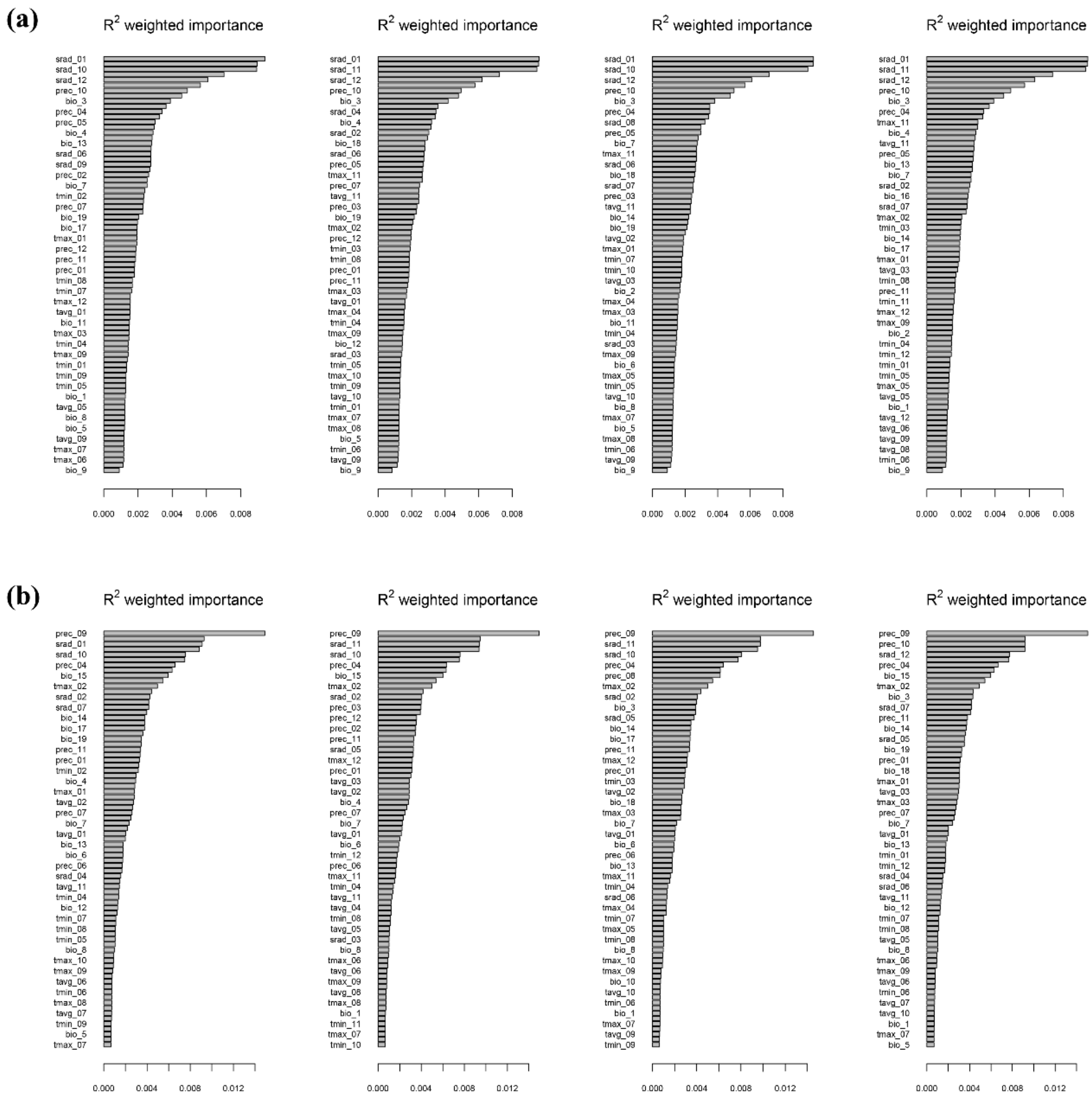

Fig. S7. Importance ranking of all environmental variables based on gradient forest (GF) analysis of 50,000 randomly selected SNPs from (a) *A. eriantha* and (b) *A. hemsleyana* (four replicates per species).

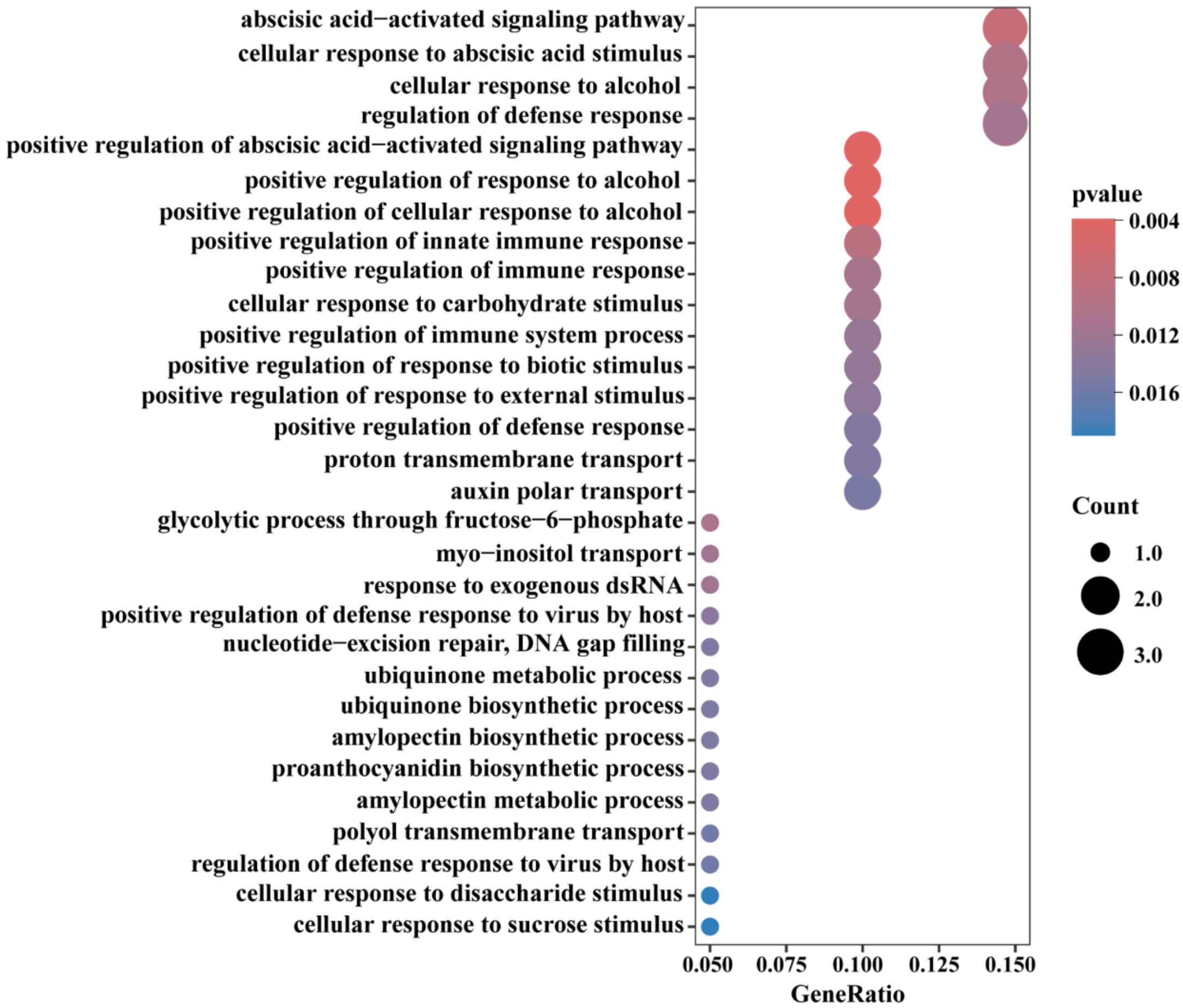

Fig. S8. GO enrichment analysis of the 41 environmental adaptation candidates, which represent the overlap between the GEA candidates (shared genes identified through genotype-environment association analyses) and selected candidates (shared genes identified through selective scans).

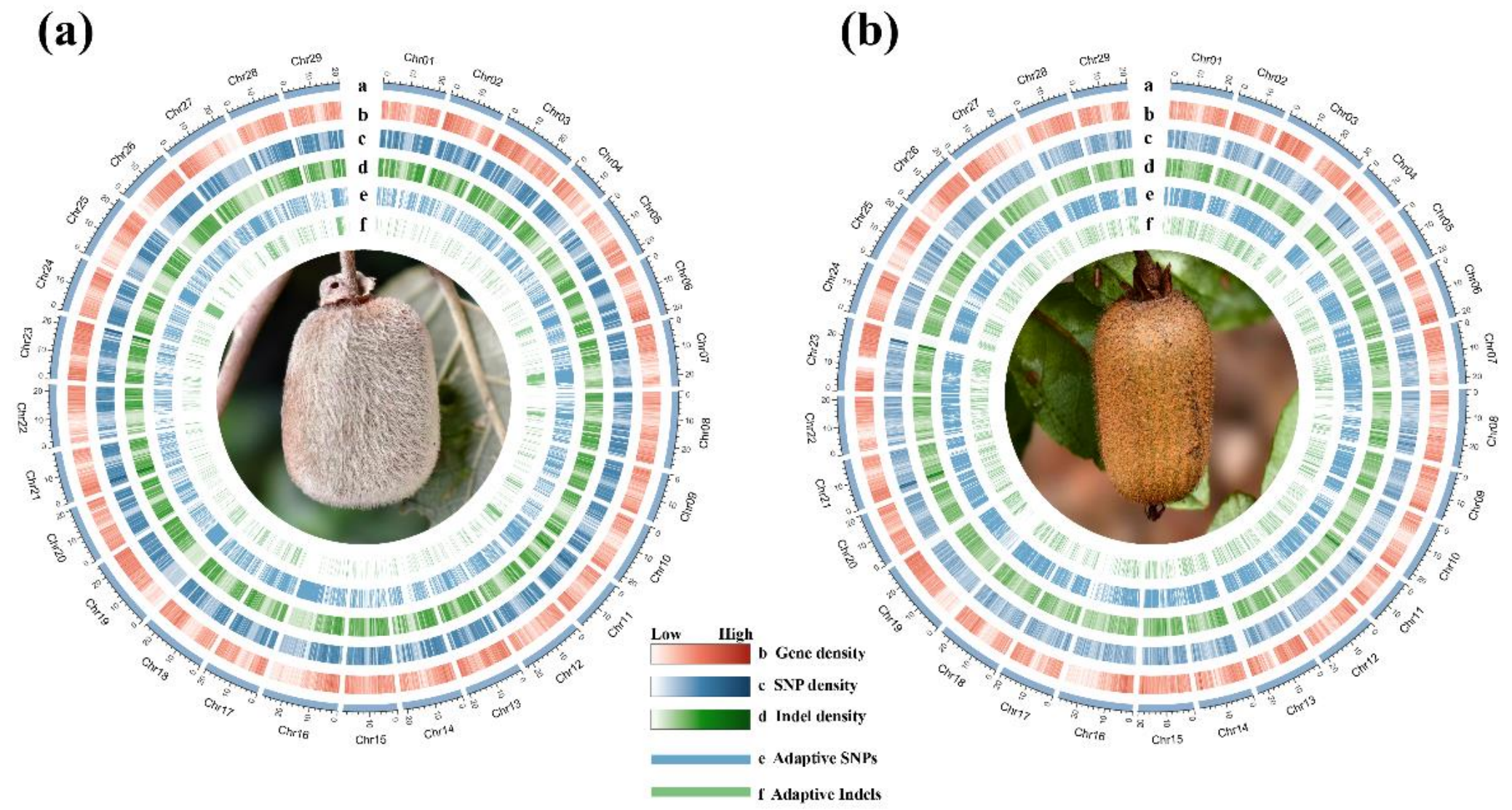

Fig. S9. Genome-wide distribution of adaptive SNPs associated with environments factors in (a) *A. eriantha* and (b) *A. hemsleyana*, respectively. Tracks from outermost to innermost are (a) Chr number, (b) gene density on the whole genome, (c) SNP density, (d) indels density, and the distribution of potentially climatic-adaptive (e) SNPs and (f) indels identified by both LFMM and RDA across the genome.
